# Redox Regulation of Endogenous Gasotransmitters in Vascular Health and Disease

**DOI:** 10.3390/ijms26189037

**Published:** 2025-09-17

**Authors:** Giang-Huong Vu, Cuk-Seong Kim

**Affiliations:** Department of Physiology & Medical Science, College of Medicine, Chungnam National University, Daejeon 35015, Republic of Korea; gianghuong.huongvu@gmail.com

**Keywords:** hydrogen sulfide, nitric oxide, carbon monoxide, reactive oxygen species, cardiovascular disease, gaseous signaling

## Abstract

Hydrogen sulfide (H_2_S), nitric oxide (NO), and carbon monoxide (CO) are now recognized as key gasotranmitters that regulate vascular function, contributing to vasodilation, angiogenesis, inflammation control, and oxidative balance. Initially regarded as toxic gases, they are produced on demand by specific enzymes, including cystathionine γ-lyase (CSE), endothelial nitric oxide synthase (eNOS), and heme oxygenase-1 (HO-1). Their activity is tightly controlled by redox-sensitive pathways. Reactive oxygen species (ROS), particularly superoxide and hydrogen peroxide, modulate gasotransmitter biosynthesis at the transcriptional and post-translational levels. Moreover, ROS affect gasotransmitter availability through oxidative modifications, including thiol persulfidation, nitrosative signaling, and carbonylation. This redox regulation ensures a tightly coordinated response to environmental and metabolic cues within the vascular system. This review synthesizes the current understanding of redox–gasotransmitter interactions, highlighting how ROS modulate the vascular roles of H_2_S, NO, and CO. Understanding these interactions provides critical insights into the pathogenesis of cardiovascular diseases and offers potential redox-targeted therapies.

## 1. Introduction

Hydrogen sulfide (H_2_S), nitric oxide (NO), and carbon monoxide (CO) are small, membrane-permeable molecules that are endogenously synthesized via distinct enzymatic pathways. Cystathionine γ-lyase (CSE) and cystathionine β-synthase (CBC) produce H_2_S through the transsulfuration pathway [1], endothelial nitric oxide synthase (eNOS) generates NO from L-arginine [2], and heme oxygenase-1 degrades heme to release CO [3]. These gaseous molecules, collectively referred to as gasotransmitters, initiate localized signaling processes within vascular cells without the need for membrane receptors or vesicular transport [4].

Although initially identified as environmental pollutants, gasotransmitters are now recognized as integral regulators of vascular function. Their physiological actions include modulation of vasomotor tone, inhibition of smooth muscle proliferation, promotion of angiogenesis, and suppression of inflammatory responses [4]. Impairments in gasotransmitter synthesis or signaling have been implicated in the pathogenesis of cardiovascular disorders such as hypertension, atherosclerosis, ischemia–reperfusion injury, and heart failure [5,6,7,8].

The biological activities of gasotransmitters are closely linked to redox homeostasis within vascular cells. Reactive oxygen species (ROS), including superoxide and hydrogen peroxide (H_2_O_2_), function as both intracellular messengers and inducers of oxidative stress. At physiological levels, ROS participate in reversible post-translational modifications such as thiol oxidation, which facilitate signaling cascades [9,10]. Under conditions of redox imbalance, excessive ROS accumulation promotes oxidative injury, disrupts endothelial function, and contributes to vascular remodeling [11,12]. Growing evidence suggests that ROS exert both direct and indirect control over gasotransmitter systems. Transcriptional and post-translational regulation of gasotransmitter-producing enzymes is modulated by redox-sensitive factors, including nuclear factor erythroid 2-related factor 2 (NRF2) and hypoxia-inducible factor-1α (HIF-1α) [13,14]. Conversely, H_2_S, NO, and CO modulate intracellular redox status by scavenging reactive species, enhancing the expression of antioxidant enzymes, and maintaining mitochondrial function. Post-translational modifications such as persulfidation, S-nitrosylation, and carbonylation further influence gasotransmitter bioavailability, stability, and receptor interaction [15,16].

The reciprocal regulation between ROS and gasotransmitters plays a central role in preserving endothelial integrity and vascular homeostasis. Under controlled redox conditions, ROS enhance NO production and vasodilatory capacity. When oxidative stress becomes excessive, NO bioactivity declines because of peroxynitrite formation and endothelial nitric oxide synthase (eNOS) uncoupling [17]. H_2_S contributes to redox balance by upregulating antioxidant defenses and detoxifying ROS, thereby counteracting endothelial dysfunction [18]. Similarly, CO modulates mitochondrial oxidative stress responses and promotes the expression of cytoprotective proteins [19]. Despite the recognition of redox–gasotransmitter interactions, several unresolved questions persist. The molecular mechanisms that determine whether ROS act as physiological regulators or pathological agents remain incompletely defined. The relative contributions of different ROS sources, such as NADPH oxidases (NOX) and mitochondria, to gasotransmitter signaling dynamics require further clarification. In addition, the spatial and temporal characteristics of gasotransmitter regulation within distinct vascular cell types are not yet fully understood.

This review presents an integrated analysis of current knowledge regarding the redox-dependent regulation of H_2_S, NO, and CO in vascular biology. Emphasis is placed on enzymatic control mechanisms, molecular interactions, and functional implications under physiological and pathological conditions. Understanding how oxidative signals shape gasotransmitter-mediated pathways may yield novel therapeutic strategies for the treatment of cardiovascular diseases.

## 2. Reactive Oxygen Species and Redox-Signaling

Reactive oxygen species (ROS) are chemically reactive molecules and free radicals derived from molecular oxygen. The primary sources of ROS within cells include mitochondrial electron transport during aerobic respiration, as well as specific enzymatic systems such as NOX, xanthine oxidase, and uncoupled nitric oxide synthase. The major representatives of ROS include superoxide (O_2_^−^), hydrogen peroxide (H_2_O_2_), and the hydroxyl radical (•OH) [20].

**Figure 1:**
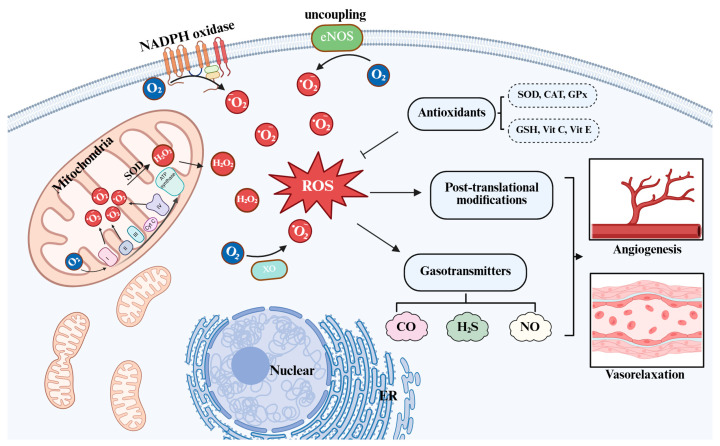
Overview of redox–gasotransmitter interplay in vascular biology. The predominant intracellular sources of ROS include mitochondrial electron transport during aerobic respiration, as well as specific enzymatic systems such as NADPH oxidases, xanthine oxidase, and uncoupled nitric oxide synthase. The major representatives of ROS include superoxide, hydrogen peroxide, and the hydroxyl radical. At physiologically relevant concentrations, these ROS can act as intracellular second messengers, modulating cell proliferation, migration, adhesion, and survival. ROS: reactive oxygen species; O_2_^−^: superoxide; H_2_O_2_: hydrogen peroxide; eNOS: endothelial nitric oxide synthase; XO: Xanthine oxidase; SOD: Superoxide dismutase; CAT: Catalase; GPx: Glutathione peroxidase; Vit C: Vitamins C; Vit E: Vitamins E; CO: Carbon monoxide; H_2_S: Hydrogen sulfide; NO: Nitric oxide. GSH: Glutathione.

Although ROS have long been associated with oxidative damage to proteins, lipids, and nucleic acids, accumulating evidence demonstrates that low to moderate concentrations of ROS participate in physiological signaling. At such concentrations, ROS can act as intracellular second messengers, influencing a wide range of cellular processes through modulation of redox-sensitive signaling pathways, including mitogen-activated protein kinases (MAPKs), phosphoinositide 3-kinase/protein kinase B (PI3K/Akt), and nuclear factor kappa B (NF-κB) [21]. In vascular endothelial cells, ROS-dependent signaling regulates cell proliferation, migration, adhesion, and survival [22] (Figure 1).

To prevent excessive ROS accumulation and consequent oxidative stress, cells are equipped with a multilayered antioxidant defense network. This defense system includes enzymatic components, such as superoxide dismutase, catalase, and glutathione peroxidase, as well as non-enzymatic antioxidants, including glutathione, thioredoxin, and vitamins C and E [23]. The interaction between ROS production and antioxidant activity maintains intracellular redox balance, also referred to as redox homeostasis. Perturbation of this balance leads to redox dysregulation and contributes to the development of vascular dysfunction and inflammation [22].

In endothelial biology, ROS generated by NOX are required for physiological responses, such as vascular endothelial growth factor (VEGF)-induced angiogenesis [24]. Systemic knockout of NOX1 has been demonstrated to compromise neovascularization during tumorigenesis in mice [25], whereas NOX4 expression is essential for exercise-induced angiogenesis in mice [26]. Conversely, uncontrolled mitochondrial ROS production has been implicated in pathological processes, including endothelial activation, increased vascular permeability, and the promotion of a pro-inflammatory phenotype [27]. Therefore, the level and origin of ROS determine whether the cellular effects are adaptive or detrimental.

The redox status of the cell, shaped by the relative abundance of ROS and antioxidants, establishes a biochemical environment that critically influences gasotransmitter function. Oxidative conditions affect both the expression of enzymes responsible for the synthesis of gasotransmitters and the stability and reactivity of the gaseous molecules themselves [20,28]. As a result, understanding ROS not only as agents of cellular stress but also as modulators of physiological signaling is fundamental for elucidating the regulation of vascular function.

## 3. Gasotransmitters

### 3.1. Biochemical Characteristics of Gasotransmitters

Hydrogen sulfide (H_2_S), nitric oxide (NO), and carbon monoxide (CO) are endogenously produced gaseous signaling molecules characterized by low molecular weight, high diffusibility, and rapid membrane permeability. These gasotransmitters are synthesized in a tightly regulated, stimulus-dependent manner, rather than being stored in vesicles. Their ability to diffuse freely across cellular and subcellular membranes enables them to function without classical receptors, thereby facilitating rapid signal transduction within vascular tissues [4].

Each gasotransmitter possesses distinct physicochemical properties. H_2_S exhibits moderate solubility in aqueous environments and can readily form polysulfides or react with metalloproteins. NO is a lipophilic, highly reactive free radical that reacts rapidly with superoxides and transition metals. CO is a non-polar, relatively stable molecule with a high affinity for heme-containing proteins, such as hemoglobin and cytochrome oxidase. These properties influence not only the spatial and temporal distribution of gasotransmitters but also their interactions with specific protein targets within endothelial and smooth muscle cells [29].

### 3.2. Functional Properties and Redox Sensitivity

The biological actions of endogenously produced H_2_S, NO, and CO are mediated through diverse molecular mechanisms, many of which are sensitive to the redox status. These gasotransmitters modulate cellular signaling via direct post-translational modifications of protein thiols, heme moieties, and reactive amino acid residues. Protein persulfidation by H_2_S modifies cysteine residues and enhances the activity of antioxidant enzymes, such as superoxide dismutase and glutathione peroxidase [30]. S-nitrosylation mediated by NO alters the functions of kinases, ion channels, and mitochondrial proteins involved in oxidative phosphorylation. CO interacts with transition metals in heme-containing proteins to regulate transcription factors and cellular respiration.

The biological half-life and reactivity of each gasotransmitter determine its spatial range of action and vulnerability to oxidative degradation. For instance, NO is rapidly inactivated by reaction with superoxide to form peroxynitrite, which contributes to endothelial dysfunction [31]. H_2_S undergoes spontaneous oxidation and conversion to sulfane sulfur species or thiosulfate [32]. CO, although relatively stable, modulates redox-sensitive pathways by inhibiting mitochondrial cytochrome oxidase and by inducing heme oxygenase via feedback mechanisms [33]. These redox interactions position the gasotransmitters as both regulators and targets of oxidative stress in the vascular system.

### 3.3. Enzymatic Biosynthesis and Redox Regulation

The synthesis of H_2_S, NO, and CO is catalyzed by specific enzymes that are tightly regulated by redox-dependent mechanisms. H_2_S is produced through the transsulfuration pathway by CSE and CBS, which utilize cysteine as a substrate [34]. The expression and activity of these enzymes are modulated by cellular redox conditions, with oxidative stress leading to post-translational inhibition or proteasomal degradation [35]. NO is synthesized from L-arginine by endothelial nitric oxide synthase (eNOS), an enzyme that requires cofactors such as tetrahydrobiopterin and flavins. Under conditions of oxidative imbalance, eNOS becomes uncoupled, resulting in superoxide generation instead of nitric oxide release [36]. CO is generated via the degradation of heme by heme oxygenase enzymes, particularly the inducible isoform heme oxygenase-1. This enzyme is upregulated in response to oxidative stress, hypoxia, and inflammatory stimuli through the activation of redox-sensitive transcription factors, including NRF2 and activator protein-1. Heme oxygenase-1 not only contributes to CO production but also exerts antioxidant and anti-inflammatory effects through biliverdin and ferritin upregulation [37].

Together, the gasotransmitters and their biosynthetic enzymes form a redox-sensitive network that dynamically integrates environmental cues to fine-tune vascular responses. The interactions between gas production, enzymatic regulation, and oxidative signaling underscore a central axis of vascular redox biology. This regulatory system enables the precise modulation of endothelial tone, angiogenic activity, and cellular defense against oxidative injury.

## 4. Hydrogen Sulfide (H_2_S)

**Figure 2:**
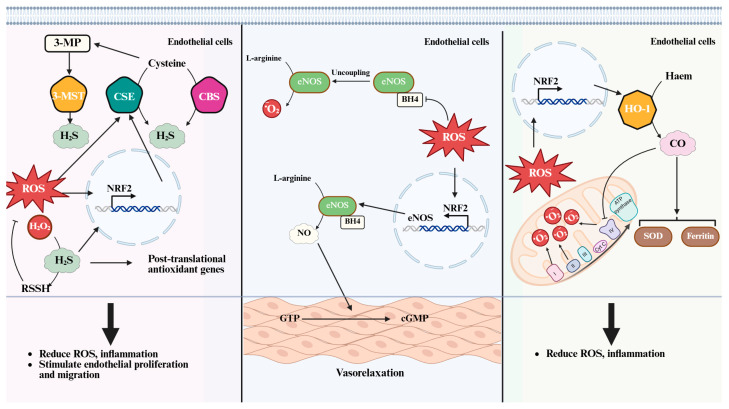
ROS-gasotransmitter crosstalk in vascular pathophysiology. ROS modulate gasotransmitter systems through both direct interactions and indirect regulatory mechanisms. The expression and activity of enzymes responsible for gasotransmitter biosynthesis are influenced by redox-sensitive transcriptional and post-translational pathways, notably involving NRF2. Each gasotransmitter contributes to vascular endothelial protection by attenuating oxidative stress, suppressing inflammatory responses, and regulating cellular metabolic processes. ROS: reactive oxygen species; O_2_^−^: superoxide; H_2_O_2_: hydrogen peroxide; eNOS: endothelial nitric oxide synthase; NRF2: Nuclear factor erythroid 2-related factor 2; CO: Carbon monoxide; HO-1: Heme oxygenase-1; SOD: Superoxide dismutase; H_2_S: Hydrogen sulfide; CSE: Cystathionine gamma-lyase; CBS: cystathionine beta-synthase; 3-MST: 3-mercaptopyruvate sulfurtransferase; 3-MP: 3-mercaptopyruvate; RSSH: Hydropersulfides; NO: Nitric oxide; BH4: Tetrahydrobiopterin; GTP: Guanosine Triphosphate; cGMP: cyclic guanosine monophosphate.

H_2_S is an endogenously produced gaseous signaling molecule that plays an essential role in the maintenance of vascular homeostasis. Within mammalian tissues, H_2_S is generated primarily via the transsulfuration pathway. The enzymes responsible for its synthesis include CSE, CBC, and 3-MST (Figure 2). These enzymes utilize substrates such homo-cysteine, L-cysteine, and their derivatives derived from diet or endogenous protein turnover [38]. Both CBC and CSE catalyze a β-replacement reaction between homocysteine and cysteine to form cystathionine and H_2_S [39]. Moreover, CSE can also convert cystathionine back into cysteine, enabling further H_2_S generation [40]. Additionally, 3-MST, a mitochondrial enzyme, generate H_2_S by transferring sulfur from 3-mercaptopyruvate to sulfurous acid, forming thiosulfate, which is subsequently reduced to H_2_S [41]. These enzymes exhibit distinct patterns of tissue distribution. CSE is considered the predominant contributor to H_2_S production in vascular endothelial and smooth muscle cells [42,43], while CBS is mainly found in the central nervous system [44,45]. Like CBS and CSE, 3-MST is present in multiple tissues, showing particularly high activity in cardiac cells, pericentral hepatocytes in the liver and the proximal tubular epithelium of the kidney [46].

The biological actions of H_2_S encompass a wide range of vascular functions. These include the promotion of endothelium-dependent vasodilation, inhibition of vascular inflammation, protection against oxidative stress, and facilitation of angiogenic signaling [47,48,49]. Mechanistically, H_2_S exerts its effects through chemical modification of protein cysteine residues, resulting in the formation of persulfide groups (-SSH) from thiol groups (-SH). Such post-translational modifications influence protein conformation and function, thereby modulating cellular processes related to redox balance, signal transduction, and metabolism. H_2_S enhances the persulfication of transient receptor potential cation channel V4 which conducts Ca^2+^ into the cytosol in aortic endothelial cells, promoting vasodilation [50]. H_2_S increased S-sulhydration of Kelch-like ECH-associated protein 1 (Keap1), facilitating the release and nuclear translocation of NRF2. This activation of NRF2 signaling has been shown to mitigate diabetes-accelerated atherosclerosis in mice [51].

The redox environment exerts a profound influence on both the biosynthesis and downstream activity of H_2_S. Exposure to moderate levels of H_2_O_2_ enhances the transcriptional activity of the CSE gene, an effect mediated by redox-sensitive transcription factors such as NRF2 [20,52]. Increased expression of CSE leads to elevated H_2_S production, which in turn contributes to antioxidant defense. Endogenous sources of H_2_O_2_, including NOX4, have been shown to modulate H_2_S levels by upregulating CSE in endothelial cells [53]. In contrast, excessive or unregulated oxidative stress may overwhelm this compensatory mechanism, leading to dysregulated H_2_S signaling and impaired vascular function.

In addition to transcriptional control, H_2_S directly interacts with reactive oxygen species. H_2_S reacts with H_2_O_2_ to form polysulfide species, which possess distinct biological activities and signaling properties. Polysulfides are capable of modifying redox-sensitive proteins through persulfidation, often leading to protective effects under oxidative conditions. The formation of such reactive sulfur species represents a crucial mechanism by which H_2_S transduces redox signals into functional cellular responses [54].

Experimental studies using both genetic and pharmacological approaches have demonstrated the protective effects of H_2_S in models of cardiovascular disease. The inhibition or deletion of cystathionine γ-lyase results in elevated blood pressure [55,56], endothelial dysfunction [57,58], and enhanced oxidative stress [59], whereas the administration of H_2_S donors improves vascular tone [60,61] and reduces inflammation [62]. Despite compelling preclinical evidence, translation into clinical applications remains limited by challenges in donor stability, delivery methods, and tissue specificity.

Collectively, H_2_S serves as a key endogenous modulator of redox signaling within the vascular system. Through both enzymatic regulation and direct molecular interactions, H_2_S integrates oxidative cues and vascular responses, contributing to the preservation of endothelial integrity and the resolution of inflammatory damage. Understanding the precise mechanisms governing H_2_S synthesis and action will provide valuable insights into the development of therapeutic strategies targeting redox imbalances in cardiovascular diseases.

## 5. Nitric Oxide (NO)

NO is a small, endogenously synthesized gas that plays a central role in regulating vascular function. The production of NO occurs through the oxidation of L-arginine by nitric oxide synthase enzymes, which exist in three major isoforms: neuronal, inducible, and endothelial. Among these, endothelial nitric oxide synthase (eNOS) is the primary source of NO in vascular endothelial cells and serves as a critical modulator of vascular tone, platelet aggregation, and endothelial integrity [63].

The biological actions of NO are mediated through both cyclic guanosine monophosphate (cGMP)-dependent and cGMP-independent pathways. Activation of soluble guanylate cyclase leads to an increase in cGMP levels, which in turn promotes vasodilation through protein kinase G signaling [64]. In addition, NO can directly modify cysteine or tyrosine residues in target proteins through S-nitrosylation and nitration, respectively. Such post-translational modifications influence protein function and are involved in the regulation of cellular processes, including apoptosis [65], cytoskeletal remodeling [66,67], and gene expression [68].

The synthesis and function of NO are tightly coupled to the redox state of the cell. Under physiological conditions, low levels of ROS enhance NO production by stimulating the expression and activity of eNOS (Figure 2). H_2_O_2_ derived from NOX4 serves as a key modulator of eNOS transcription and phosphorylation, contributing to nitric oxide-dependent angiogenic responses [69]. However, when oxidative stress becomes excessive, the reaction between NO and superoxide anions generates peroxynitrite, a reactive nitrogen species that reduces NO bioavailability and promotes cellular injury [70].

Redox-dependent modifications of eNOS further complicate NO regulation. In the absence of sufficient cofactors such as tetrahydrobiopterin or L-arginine, eNOS becomes uncoupled and begins to produce superoxide instead of NO. This shift not only reduces the vasodilatory capacity of NO, but also contributes to the amplification of oxidative stress [36]. Furthermore, high concentrations of nitric oxide, often observed during inflammatory activation of inducible NO synthase, can exacerbate nitrosative stress [71] and mitochondrial dysfunction [36,72,73].

NO plays a dual role in vascular biology, functioning as both a protective and potentially harmful mediator depending on the context and concentration. At low concentrations, NO promotes endothelial health by maintaining vascular tone [74], inhibiting leukocyte adhesion [75], and suppressing smooth muscle proliferation [76]. At high concentrations or under conditions of oxidative imbalance, NO contributes to vascular pathology through the formation of cytotoxic species and disruption of redox-sensitive signaling pathways [77,78,79].

Therapeutic strategies aimed at restoring NO bioavailability have demonstrated beneficial effects in models of hypertension [80], atherosclerosis [81], and ischemia–reperfusion injury [82]. Pharmacological interventions targeting nitric oxide synthesis, such as L-arginine supplementation, tetrahydrobiopterin stabilization, or eNOS activation, offer potential avenues for modulating the redox balance in cardiovascular diseases [83,84]. Nonetheless, the dual nature of NO signaling necessitates precise control of both its generation and its interaction with the redox environment.

## 6. Carbon Monoxide (CO)

CO is an endogenously generated gaseous molecule that plays a regulatory role in vascular physiology and cellular homeostasis. Although carbon monoxide is a well-known toxic gas at high concentrations because of its strong affinity for hemoglobin, at physiological levels it functions as an important signaling molecule in various tissues, including the vascular system [85]. CO is produced during the degradation of heme, a process catalyzed by heme oxygenase enzymes. Two main isoforms of heme oxygenase have been identified: the inducible form, heme oxygenase-1, and the constitutive form, heme oxygenase-2. Among these, heme oxygenase-1 is the primary source of stress-inducible CO in vascular cells [86].

The biological effects of CO are diverse and context-dependent. Within the vascular system, CO modulates vasodilation [87], inhibits platelet aggregation [88], attenuates leukocyte adhesion, and suppresses pro-inflammatory signaling [89]. The vasodilatory action of CO is mediated in part through the activation of soluble guanylate cyclase, leading to an increase in cGMP levels. Additionally, CO interacts with heme-containing proteins and iron–sulfur clusters, thereby influencing cellular respiration, mitochondrial function, and redox-sensitive metabolic pathways [90,91].

The expression of heme oxygenase-1 is tightly regulated by oxidative stress, hypoxia, and other inflammatory stimuli (Figure 2). Induction of heme oxygenase-1 is mediated by redox-sensitive transcription factors, including NRF2, activator protein-1, and HIF-1α. In vascular endothelial cells, H_2_O_2_ acts as an upstream signal that activates these transcription factors, resulting in increased heme oxygenase-1 expression and subsequent carbon monoxide production. The NRF2-heme oxygenase-1 axis serves as a critical protective mechanism against oxidative injury, promoting antioxidant gene expression, and enhancing cellular resistance to stress [92,93,94].

In addition to transcriptional regulation, CO influences cellular redox balance through both direct and indirect mechanisms. CO reversibly inhibits mitochondrial complex IV activity, thereby modulating mitochondrial ROS production under stress conditions [95]. CO also regulates the activity of mitochondrial hemoproteins, including cytochrome c oxidase, which allows CO to reduce excessive ROS generation while preserving essential mitochondrial signaling. Furthermore, CO promotes the expression of antioxidant enzymes, including superoxide dismutase and ferritin, thereby contributing to the overall redox defense of the endothelium [96].

Experimental models have demonstrated the protective effects of CO in various vascular disease contexts, including hypertension [97] and ischemia–reperfusion injury [98]. CO exerts cardioprotective effects through its interaction with mitochondrial. This is evidenced by its ability to active mitochondrial K_ATP_ channel and inhibit the opening of the mitochondrial permeability transition pore [99]. In a rat animal model of cardiac ischemia–reperfusion, after coronary occlusion, CO exposure markedly reduced the ratio of infarct areas to risk areas and attenuated the infiltration of inflammatory cells, specifically macrophages and monocytes into infarct areas [98]. Administration of CO donors or pharmacological inducers of heme oxygenase-1 has been shown to improve endothelial function, reduce vascular inflammation, and limit oxidative damage [100]. Despite these promising findings, clinical translation remains challenging because of safety concerns, dose-dependent toxicity, and the need for controlled delivery systems.

In the context of redox regulation, CO functions both as a product and a modulator of oxidative stress. Its generation is triggered by oxidant exposure, and in turn, CO restores the redox balance through cytoprotective, anti-inflammatory, and antioxidant mechanisms. The role of CO in vascular homeostasis underscores the importance of heme oxygenase-1 as a therapeutic target and highlights the potential of gasotransmitter-based interventions in cardiovascular disease [29,101].

## 7. Comparative Analysis of Gasotransmitters

H_2_S, NO, and CO represent a triad of endogenous gaseous signaling molecules with distinct chemical properties, overlapping biological functions, and convergent regulation by the cellular redox environment. Although each molecule is produced by different enzymatic pathways and exerts specific molecular actions, all three serve as critical modulators of vascular physiology and redox signaling.

The biosynthetic origin of each gasotransmitter is tightly linked to specialized enzymes. H_2_S is produced from cysteine via cystathionine γ-lyase, cystathionine β-synthase, and 3-mercaptopyruvate sulfurtransferase. NO is synthesized from L-arginine through nitric oxide synthase isoforms, while CO is generated through the catabolism of heme oxygenase-1 and heme oxygenase-2. Although these enzymatic systems differ in their substrate requirements and expression patterns, they are all subject to regulation by oxidative stress and redox-sensitive transcription factors [20].

These three gasotransmitters exert partially overlapping physiological functions in the vascular system. Each molecule promotes vasodilation, inhibits vascular inflammation, and maintains endothelial integrity (Table 1). NO is particularly potent in activating soluble guanylate cyclase and stimulating cyclic guanosine monophosphate signaling [64]. H_2_S influences vascular tone through KATP channel opening and persulfidation of signaling proteins [102], while CO modulates vascular tone via both soluble guanylate cyclase activation and mitochondrial metabolic regulation [19].

Differences in redox sensitivity and interactions with ROS further distinguish these molecules. NO reacts rapidly with superoxide to form peroxynitrite, a highly reactive nitrogen species that contributes to endothelial dysfunction under pathological conditions [70]. H_2_S reacts with H_2_O_2_ to form polysulfides, which exert protective persulfidation on protein thiols [54]. CO modulates mitochondrial ROS production by reversibly inhibiting cytochrome c oxidase and enhancing antioxidant defense through the upregulation of ferritin and superoxide dismutase expression [37,96].

The redox–gasotransmitter axis forms a tightly integrated feedback system in endothelial cells. Oxidative stress upregulates gasotransmitter synthesis via transcriptional and post-translational mechanisms. In turn, gasotransmitters act to restore redox balance through antioxidant, anti-inflammatory, and cytoprotective actions. However, the response is context-dependent. For example, NO becomes detrimental when produced in excess under inflammatory conditions, whereas H_2_S and CO are more consistently associated with protective effects against oxidative damage [16,103,104].

Despite these shared features, the temporal dynamics and spatial distribution of gasotransmitter signaling differ significantly. NO is produced rapidly and acts within seconds, making it suitable for acute vasodilatory responses. H_2_S and CO exhibit more sustained effects through transcriptional regulation, mitochondrial modulation, and protein modification. The compartmentalization of their respective enzymes also contributes to signal specificity and tissue selectivity [105].

Understanding the similarities and differences among H_2_S, NO, and CO provides valuable insights into the integration of redox and gas signaling in vascular biology. The interplay between these gasotransmitters may represent a coordinated network rather than isolated signaling events. Further research is required to determine whether synergistic or compensatory mechanisms exist among them, particularly under pathological conditions such as hypertension, atherosclerosis, and ischemia.

## 8. Conclusions and Future Perspectives

The redox-dependent regulation of H_2_S, NO, and CO presents a promising framework for the development of novel therapeutic strategies targeting cardiovascular diseases. Each of these gasotransmitters exerts protective effects on the vascular endothelium by modulating oxidative stress, inflammation, and cellular metabolism. Given their central role in maintaining vascular homeostasis, pharmacological manipulation of gasotransmitter signaling holds considerable potential for restoring redox balance and preventing disease progression in oxidative stress-driven pathologies.

NO donors have long been utilized in clinical practice for the management of angina and hypertension [106]. Organic nitrates, such as nitroglycerin and isosorbide dinitrate, provide short-term vasodilatory benefits, although their chronic administration is limited by the development of tolerance and oxidative side effects [107]. To address these challenges, newer compounds targeting eNOS activation or tetrahydrobiopterin stabilization are under investigation, aiming to enhance endogenous NO production under physiological conditions. Combination of propranolol and the BH4 precursor 5-methyltetrahydrofolate demonstrated superipor efficacy in reducing hepatic venous pressure gradient in patients with liver cirrhosis compared to propranolol monotherapy [108]. Furthermore, a 2025 clinical trial involving individuals with peripheral artery disease revealed that co-administration of L-citrulline and BH4 improved the absolute claudication distance as an indirect marker of improved vascular function [109].

H_2_S-based therapeutics represent a rapidly expanding area of translational research. Various slow-releasing H_2_S donors, including GYY4137 and SG1002, have demonstrated efficacy in preclinical models of atherosclerosis, myocardial infarction, and hypertension [110,111,112,113,114]. These compounds not only induce vasorelaxation but also upregulate antioxidant enzymes, inhibit leukocyte adhesion, and preserve mitochondrial function. Current challenges include achieving tissue-specific delivery, optimizing pharmacokinetics, and preventing overexposure to reactive sulfur species. Therefore, Xian Zheng and colleagues developed a photothermal H_2_S nanogenerator (PSA@ADT-OH), composed of a perylene-cored photothermal agent (PSA) and the H_2_S donor ADT-OH. This system exhibits high shear-resistant targeting to thrombi and enables sustained H_2_S release, promoting antiplatelet aggregation, and vascular healing [115].

CO therapy has garnered attention for its anti-inflammatory and cytoprotective effects. While inhaled carbon monoxide poses toxicity risks, low-dose administration through controlled delivery systems has shown therapeutic potential in animal models of vascular injury and transplant rejection [116,117]. Pharmacological inducers of heme oxygenase-1, such as hemin and metalloporphyrins, are being explored to stimulate endogenous carbon monoxide production [118]. In addition, carbon monoxide-releasing molecules (CORMs) offer a promising approach for site-selective and dose-regulated delivery [119]. In a rat model of hemorrhagic shock, pretreatment with CORM2 inhibits inflammation [120], while luminal administration of CORM3 mitigates ischemia–reperfusion injury in rats following intestinal transplantation [121]. Nonetheless, further studies are required to assess long-term safety, dosage precision, and off-target effects.

Future therapeutic strategies are expected to leverage the synergistic properties of gasotransmitters and their interactions with the redox environment. Combination therapies targeting multiple gasotransmitter pathways, or co-administering gas donors with antioxidants may offer enhanced protection against complex vascular diseases. Nanoparticle-based delivery platforms and gene therapy approaches are also being developed to improve the bioavailability, cell-type specificity, and on-demand release of gasotransmitters in vivo.

Advances in molecular imaging and omics technologies have provided powerful tools for monitoring redox–gasotransmitter signaling dynamics at the cellular level. Single-cell transcriptomics, real-time redox biosensors, and spatial proteomics enable high-resolution mapping of gasotransmitter effects across different vascular cell types and disease stages. These technologies are expected to facilitate the identification of redox-sensitive biomarkers and the refinement of personalized therapeutic strategies.

The integration of gasotransmitter biology into the broader landscape of redox medicine offers an opportunity to redefine the therapeutic paradigms for cardiovascular and inflammatory diseases. Continued investigation into the molecular mechanisms, temporal dynamics, and tissue specificity of gasotransmitter action will be essential for translating preclinical findings into safe and effective clinical applications. Collaboration among basic scientists, pharmacologists, and clinicians will be critical for bridging experimental insights with therapeutic innovation.

## Figures and Tables

**Table 1:** Features of gasotransmitters.

Feature	Hydrogen Sulfide	Nitric Oxide	Carbon Monoxide
**Biosynthetic enzyme**	CSE, CBS, 3-MST	data eNOS, nNOS, iNOS	HO-1, HO-2
**Primary** **action**	K^+^ channel opening, thiol persulfidation	data sGC activation, S-nitrosylation	sGC activation, mitochondrial regulation
**Redox** **interaction**	data Reacts with H_2_O_2_ →polysulfides	Reacts with O_2_^−^ →ONOO^−^	Inhibits mitochondrial ROS
**Antioxidant** **effect**	data Upregulates antioxidant proteins	Scavenges ROS, but forms RNS	Induces SOD, ferritin
**Vascular role**	data Vasodilation, angiogenesis	Vasodilation, anti-inflammation	Vasodilation, anti-apoptosis
**Clinical** **challenges**	Donor stability, specificity	Dual role, oxidative vulnerability	Delivery method, toxicity

## Data Availability

Not applicable.
